# Responses of *AG1* and *AG2* QTL introgression lines and seed pre-treatment on growth and physiological processes during anaerobic germination of rice under flooding

**DOI:** 10.1038/s41598-020-67240-x

**Published:** 2020-06-23

**Authors:** Satyen Mondal, M. Iqbal R. Khan, Frederickson Entila, Shalabh Dixit, Pompe C. Sta. Cruz, M. Panna Ali, Barry Pittendrigh, Endang M. Septiningsih, Abdelbagi M. Ismail

**Affiliations:** 1 International Rice Research Institute, DAPO Box 7777, Metro Manila, Philippines; 2 Bangladesh Rice Research Institute, Gazipur 1701, Bangladesh; 3 University of the Philippines Los Baños, College 4031 Laguna, Philippines; 4 Jamia Hamdard, New Delhi 110062, India; 5 Michigan State University, East Lansing, MI USA; 6 Texas A&M University, College Station, TX USA

**Keywords:** Carbohydrates, Chemical modification, Enzyme mechanisms, Enzymes, Plant breeding, Plant cell biology, Plant ecology, Plant genetics, Plant physiology, Plant stress responses

## Abstract

Rice seeds germinating in flooded soils encounter hypoxia or even anoxia leading to poor seed germination and crop establishment. Introgression of *AG1* and *AG2* QTLs associated with tolerance of flooding during germination, together with seed pre-treatment via hydro-priming or presoaking can enhance germination and seedling growth in anaerobic soils. This study assessed the performance of elite lines incorporating *AG1, AG2* and their combination when directly seeded in flooded soils using dry seeds. The QTLs were in the background of two popular varieties PSB Rc82 and Ciherang-Sub1, evaluated along with the donors Kho Hlan On (*AG1*) and Ma-Zhan Red (*AG2*) and recipient parents PSB Rc82 and Ciherang-Sub1. In one set of experiments conducted in the greenhouse, seedling emergence, growth, and carbohydrate mobilization from seeds were assessed. Metabolites associated with reactive oxygen species (ROS) scavenging including malondialdehyde (MDA) as a measure of lipid peroxidation, ascorbate, total phenolic concentration (TPC), and activities of ROS scavenging enzymes were quantified in seeds germinating under control (saturated) and flooded (10 cm) soils. In another set of experiments conducted in a natural field with 3–5 cm flooding depths, control and pretreated seeds of Ciherang-Sub1 introgression lines and checks were used. Flooding reduced seedling emergence of all genotypes, though emergence of *AG1* + *AG2* introgression lines was greater than the other AG lines. Soluble sugars increased, while starch concentration decreased gradually under flooding especially in the tolerant checks and in *AG1* + *AG2* introgression lines. Less lipid peroxidation and higher α-amylase activity, higher ascorbate (RAsA) and TPC were observed in the tolerant checks and in the *AG1* + *AG2* introgression lines. Lipid peroxidation correlated negatively with ascorbate, TPC, and with ROS scavengers. Seed hydro-priming or pre-soaking increased emergence by 7–10% over that of dry seeds. Introgression of *AG2* and *AG1* + *AG2* QTLs with seed pretreatment showed 101–153% higher emergence over dry seeds of intolerant genotypes in the field. Lines carrying *AG1* + *AG2* QTLs showed higher α-amylase activity, leading to rapid starch degradation and increase in soluble sugars, ascorbate, and TPC, together leading to higher germination and seedling growth in flooded soils. Seed hydro-priming or pre-soaking for 24 h also improved traits associated with flooding tolerance. Combining tolerance with seed management could therefore, improve crop establishment in flooded soils and encourage large-scale adoption of direct seeded rice system.

## Introduction

Farmers usually encounter flooding or waterlogging immediately after direct seeding of rice as a consequence of heavy rain after sowing in rainfed areas or poorly leveled fields even in irrigated areas. Flooding during seed germination and early seedling growth usually leads to poor crop establishment and low yield^1^. This is because of the high sensitivity of rice to anaerobic conditions during germination and early seedling growth^1,2^. Rice cultivars tolerant of anaerobic condition during germination are therefore required, when farmers are currently shifting from transplanting to direct-seeding methods of crop establishment^3^. Unlike other cereal crops like wheat and corn, once the crop is established, rice can grow in waterlogged and flooded soils because of its ability to develop aerenchyma tissue that facilitate aeration of roots and the rhizosphere^4–6^.

Seeds germinating in flooded soils may encounter hypoxia or even anoxia in severe cases, which could prevent germination and subsequent growth as most of the enzymes involved in the breakdown and mobilization of stored carbohydrates and/or in oxidative pathways to generate energy for growing embryos become non-functional^7–10^. Evolutionarily, germinating rice seeds acquired adaptive mechanisms to cope with oxygen deficiency in flooded soil, one of which is the ability of the coleoptile to emerge and grow quickly to keep contact with air^1^. It has been reported that coleoptiles elongate faster under low oxygen than in the air and that considerable genetic variation was observed in elongation ability within the cultivated rice gene pool^11,12^. As they come in direct contact with air, elongating coleoptiles act as snorkels to facilitate exchange of gases in waterlogged or flooded soils and maintain adequate aeration in the growing embryo. Another adaptive strategy for germinating seeds under anaerobic conditions is the primary shift from aerobic to anaerobic metabolism to generate the energy needed to sustain growth of the germinating embryo^1,13^.

In direct-seeded rice (DSR) systems, poor seedling establishment is a major constraint, where fast seed germination and seedling growth became key determinants of successful crop establishment^14^. Seed pre-treatment such as priming may enhance germination and seedling growth in DSR systems in flooded soils. Additionally, it may also improve the breakdown of stored carbohydrates by accelerating enzymatic activities of starch catabolic enzymes^15^. In such pre-treatment, seed pre-soaking could help improve germination rate, accelerate seedling growth, increase dry matter production, and improve grain yield^16,17^. Seed priming treatment resulted in better seed germination and establishment in rice (*Oryza sativa*)^15,18,19^, wheat (*Triticum aestivum*)^20^, maize (*Zea mays*)^21^, black pepper (*Piper nigrum*)^22^, and sunflower (*Helianthus annuus*)^23^. Seed hydro-priming involves soaking of seeds for a particular period, typically 24 hours with aeration and re-drying back to original moisture content (14%)^15,16^, was also reported to enhance amylase activity during germination, leading to faster seedling emergence and better vigor under stress^17,24,25^, which also help to improve weed competitiveness early in the season^26,27^.

Rice genotypes that are known for their tolerance of anaerobic conditions during germination (anaerobic germination, AG) can break down starch to soluble sugars under hypoxic conditions during germination by switching to anaerobic metabolism^1,28^, with the upregulation of anaerobic fermentation enzymes especially pyruvate decarboxylase and alcohol dehydrogenase. This pathway provides energy for successful seed germination and coleoptile elongation in flooded soils^29–32^. Under such condition, glucose supply from starch degradation, mainly through the action of starch catabolic enzymes like α-amylase, allows ATP production through fermentative metabolism^1,33^, providing energy for the germinating embryos to elongate its coleoptile and make contact with air or the better aerated upper water layers^1^.

Production of excessive reactive oxygen species (ROS) is a hallmark of abiotic stresses^34^. However, various protective mechanisms have been reported to cope with abiotic stress condition. Among these are non-enzymatic antioxidants such as reduced-form glutathione (GSH), ascorbic acid and phenols. Enzymatic antioxidants such as superoxide dismutase (SOD), catalase (CAT), ascorbate peroxidase (APX), and glutathione reductase (GR) are important for maintaining the redox balance of stressed cells^1^ by scavenging excess ROS under stress conditions^35–37^.

Identification of novel QTLs for tolerance of anaerobic conditions during germination from diverse germplasm is essential to capture the genetic complexity of the trait, to unravel the biochemical, physiological and molecular basis of tolerance and to provide QTL targets for marker-assisted breeding^12^. Donors for AG tolerance during seed germination have been identified after screening approximately 8000 accessions at the International Rice Research Institute (IRRI), including landraces such as Khaiyan (Bangladesh), Khao Hlan On (aromatic tall Japonica upland landrace, Myanmar), and Ma-Zhan red (Indica, China)^38–40^. Later on, bi-parental crosses were made with popular varieties to develop breeding lines to enhance germination under AG as well as promising genotypes with improved phenotypic and yield traits^38,39^.

*AG1* and *AG2* quantitative trait loci (QTLs) identified before, had positive effects for increased flooding tolerance during seed germination stage and are being used to develop improved rice breeding lines^1,10,12,40,41^. Exploration of additional QTLs for tolerance of anaerobic conditions during germination from diverse germplasm is essential to unravel the biochemical, physiological and molecular basis of tolerance, and to simultaneously provide additional QTL targets for marker-assisted breeding^12^.

The *AG1* QTL was mapped using a population developed from a cross of the tolerant landrace Khao Hlan On (KHO) and IR64, a non-aromatic semi-dwarf Indica lowland modern variety, which is sensitive to flooding during germination^38,39^. Subsequently, five QTLs were identified, with the large effect *qAG-9-2* (*AG1*) on the long arm of chromosome 9, with a LOD score of 20.34, explaining 33.5% of the variation for this trait. *AG1* was fine-mapped to a ~50 kb region on chromosome 9 and trehalose-6-phosphate phosphatase (*OsTPP7*) gene has been functionally validated as the gene underlying this QTL^42^.

Development of NILs for another major QTL, *qAG7.1* (*AG2*), was initiated and reported that introgression lines harboring homozygous alleles of Ma-Zhan Red in the region of *qAG7.1* have significantly higher emergence (48%) compared to the intolerant checks^12^. The two QTLs, *AG1 and* AG2 were later incorporated into different genetic backgrounds, showing no negative effects on seed physiology, while improving AG tolerance^7^.

The present study was conducted to assess the individual and interactive effects of incorporating *AG1*, *AG2* and their combination in the background of two high yielding rice varieties, Ciherang-Sub1 and PSB Rc82, both in the field and under greenhouse conditions. The study attempted to elucidate the physiological and agronomical traits associated with flooding tolerance during germination under these conditions; and to evaluate the effectiveness of seed pre-treatment before seeding on enhancing germination and crop establishment, and subsequent grain yield in natural fields.

## Results

### Seedling emergence and growth of rice seedlings during germination under flooding

**Figure 1:**
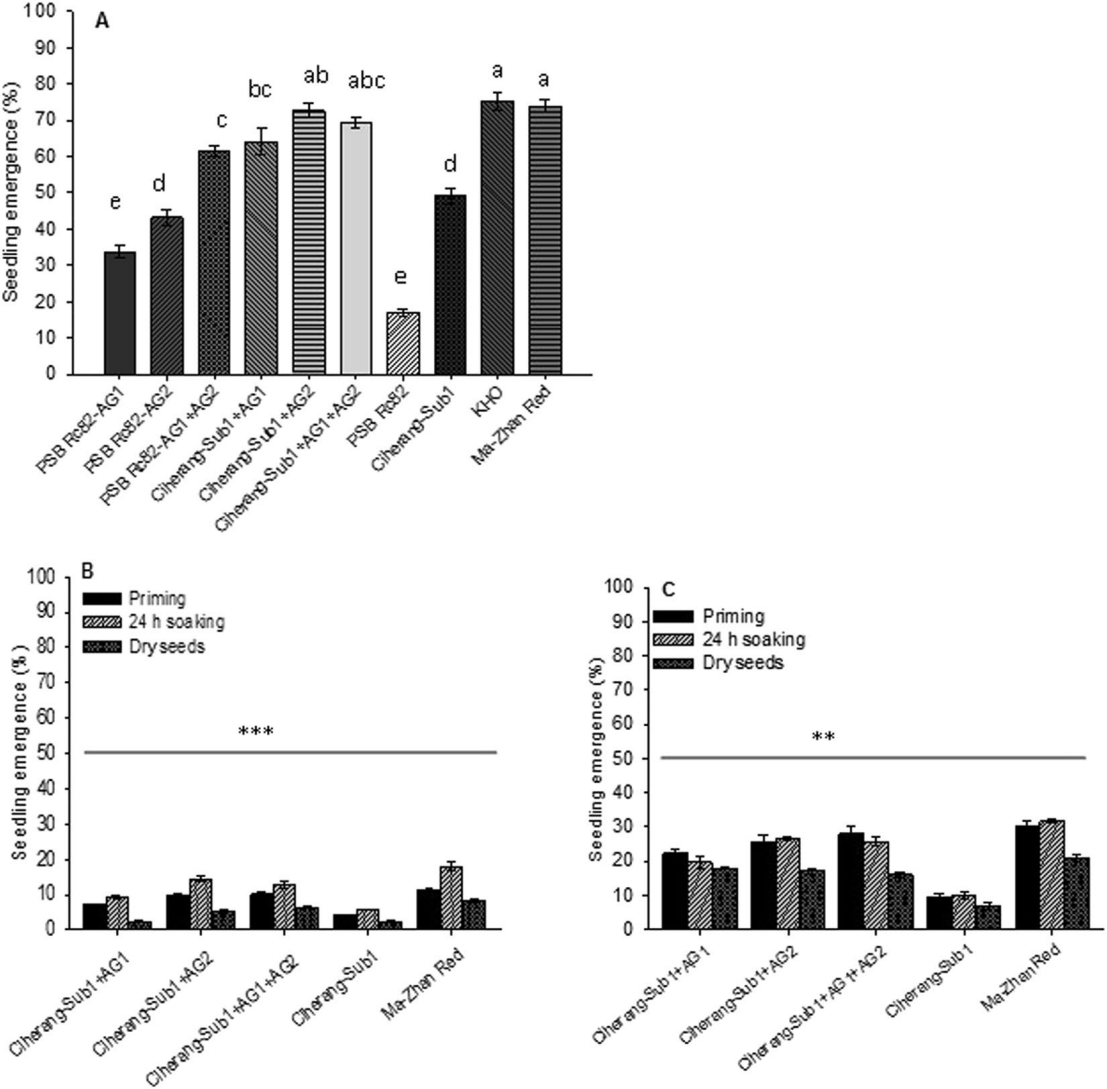
Seedling emergence (%) of AG introgression lines and checks at 21 days after seeding in greenhouse (**A**) under 10 cm water depth of flooding; and in the field (**B**,**C**) using pre-treated seeds under flooding depth of 3–5 cm during 2016 wet season (**B**) and 2017 dry season (**C**). Vertical bars indicate mean ± SE (n = 60 for the greenhouse trial; and n = 90 for field trial). Bars bearing different lower case letters indicate significant differences at p < 0.001. **significant at p < 0.01, *** significant at p < 0.001.

Seedling emergence of the tolerant and intolerant rice genotypes was similar under saturated (control) conditions, but significantly affected by flooding both in the greenhouse and under field conditions. However, emergence was lower (by over 50%) in the field than in the greenhouse (Fig. 1). In field experiments, seed soaking for 24 h significantly improved emergence of all genotypes under flooded condition compared to dry seeding (Fig. 1B,C). A significantly highest emergence (~70%) was found for Ciherang-Sub1-AG2 and Ciherang-Sub1-AG1-AG2 (*P* < 0.001) and lowest for the sensitive genotype PSB Rc82 (~16%). Seed pre-treatment increased emergence by 7–10%; and the emergence of the lines containing *AG2* and *AG1-AG2* QTLs increased by 101–153% compared with the sensitive genotype Ciherang-Sub1, in 2016 WS, and by 128–211% in 2017 DS when seeds were not treated.

**Figure 2:**
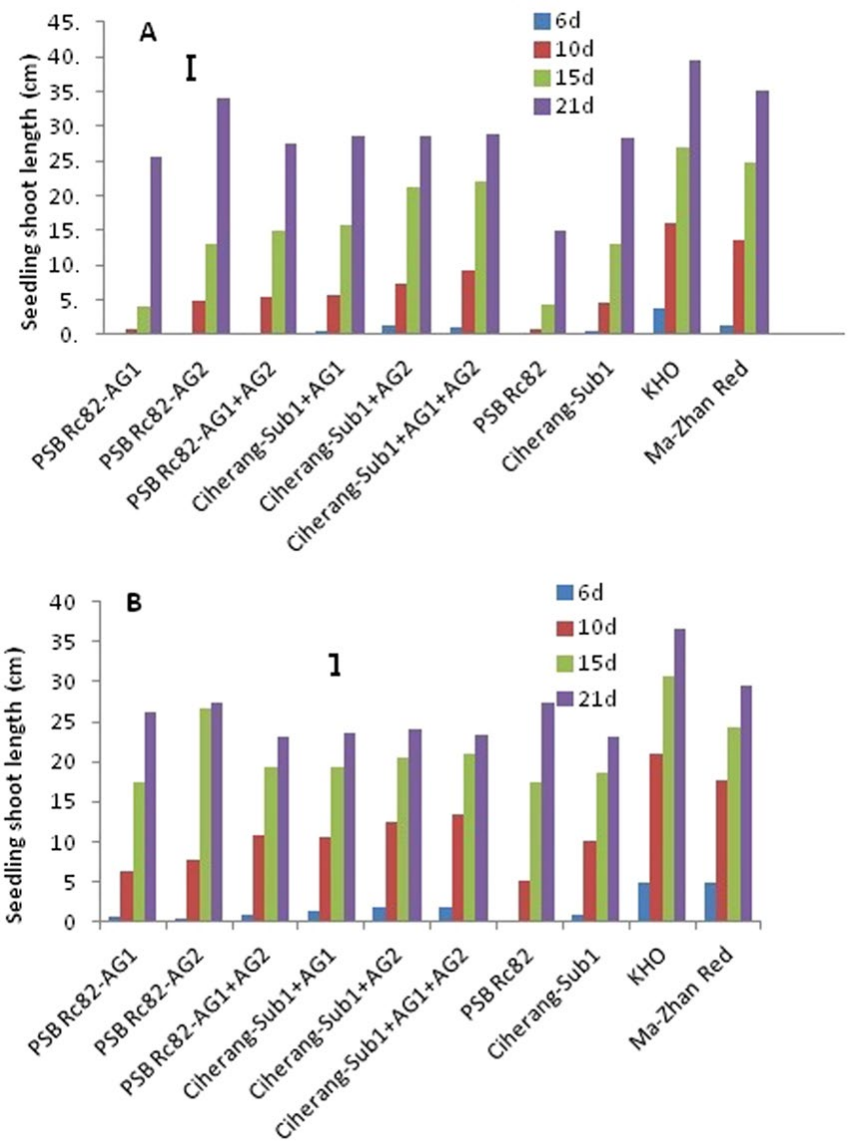
Seedling shoots length within 21days after sowing under 10 cm water depth of flooding (**A**) and under control condition (**B**) in the greenhouse experiment. Vertical bars indicate HSD_0.05_ (n = 60).

Among the ten genotypes evaluated in the greenhouse experiments, eight genotypes showed relatively higher seedling shoot length above water surface at 15 days after sowing (DAS), than PSB Rc82 and PSB Rc82-AG1 (Fig. 2). Under flooded conditions, shoot length of the tolerant introgression lines was almost double compared to that of the sensitive PSB Rc82 at 21 DAS (Fig. 2A). Under control conditions, all genotypes including Ciherang-Sub1 and PSB Rc82 showed similar shoot length, but the tolerant checks KHO and Ma-Zhan Red were relatively taller at 21 DAS (Fig. 2B). Similar trends were also observed during earlier stages (6, 10 and 15 DAS). Seedling shoot length was higher under flooding compared to the control except for PSB Rc82. Among the six-introgression lines, PSB Rc82-AG2 showed the highest shoot elongation, while the intolerant check PSB Rc82 showed the lowest elongation (Fig. 2).

**Figure 3:**
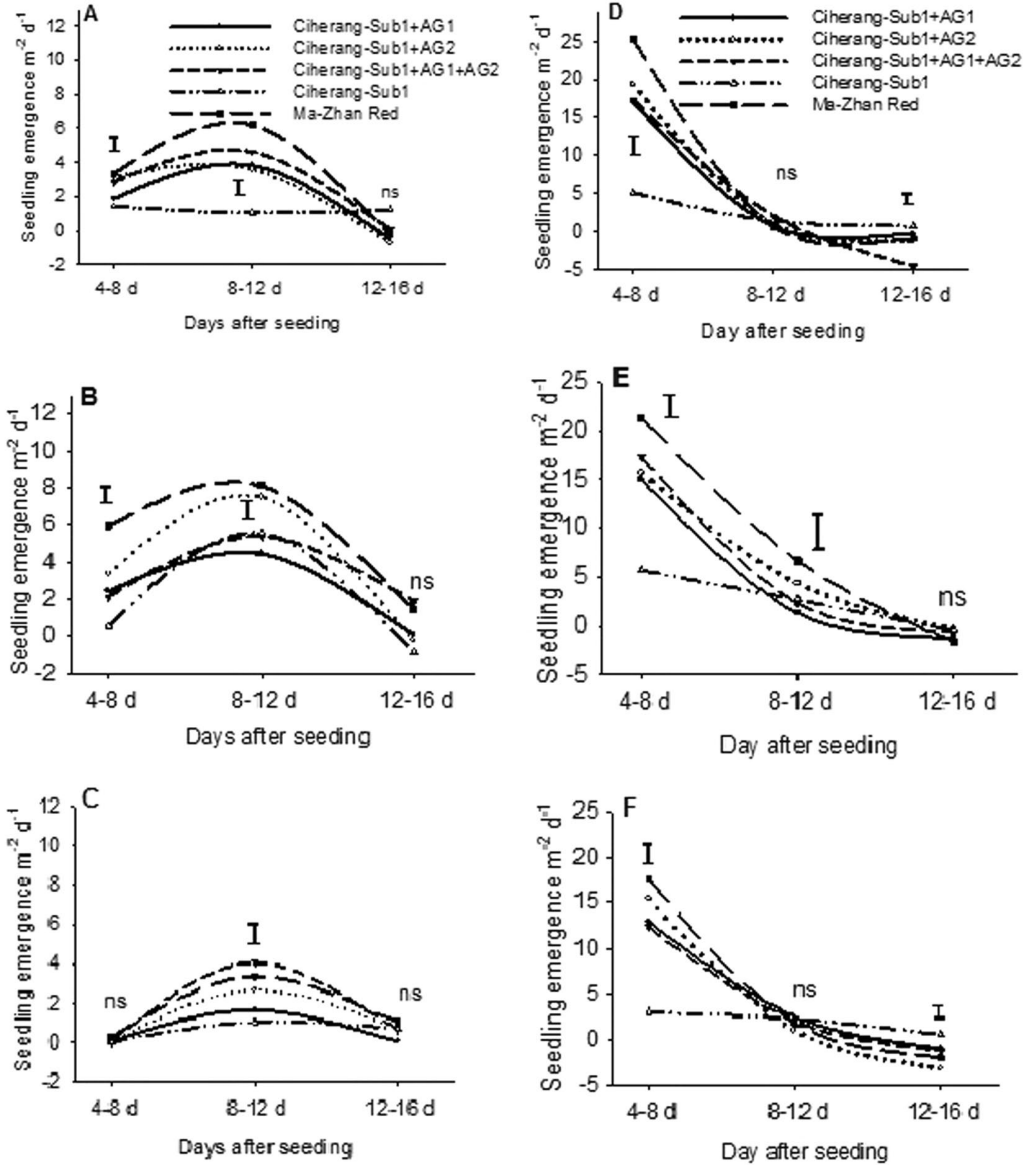
Seedling emergence rate of AG lines under field condition with pretreated seeds and flooding at 3–5 cm water depth, during the wet season of 2016 (**A–C**); (**A**) priming; (**B**) 24 h soaking; (**C**) dry seeds) and dry season 2017 (**D**–**E**); (**D**) priming; (**E**) 24 h soaking; (**F**) dry seeds). Vertical bars indicate LSD _0.05_ (n = 90).

In field experiments, across seed pretreatments, seedling emergence under flooding was significantly higher in AG-introgression lines (*P* < 0.001), and seed pre-treatment accelerated seedling emergence until 12 DAS (Fig. 3). Among the AG-lines, Ciherang-Sub1-AG2 and Ciherang-Sub1-AG1-AG2, along with the positive check Ma-Zhan Red, showed higher emergence rates across the days, seed pre-treatments, and seasons. Introgression lines carrying *AG2* and *AG1-AG2* QTLs showed 56–147% and 104–294% increase in emergence rate in flooded soil, respectively, compared with the recurrent non-introgression line. Moreover, seeds soaking for 24 h or hydro-priming showed enhanced seedling emergence over dry seeds in early seedling establishment stage, especially at the first 8 DAS, though when using dry seeds emergence increased between 8–12 DAS under flooding (Fig. 3). Emergence was steady or decreased at 12 DAS in flooded condition and at 8 DAS under control conditions.

Seedling height, plant dry-weight, and leaf area were significantly greater in AG-introgression lines (*P* < 0.05) compared to Ciherang-Sub1 under flooding (data not shown). However, 24 h pre-soaking and hydro-priming resulted in a better seedling emergence, seedling growth, and seedling height. Seedling emergence was higher in the dry season than the wet season. Correlations of emergence with seedling vigor index (SVI), leaf area, and plant dry-weight were strong and positive at 14 and 21 DAS (Table 1).

**Table 1:** Correlation coefficients for the association of emergence and different growth parameters, using pre-treated seeds sown and flooded with 3–5 cm for 21 DAS, in the dry season 2017 field experiment (n = 60; df = 9).

Parameter	Correlation Coefficient (r)								
	Emergence	SVI	LA Seedling^−1^		LW	SLA		LAI	Plant DW
			14d	21d	21d	14d	21d	21d	14d
SVI	0.96***								
LA Seedling^−1^ (14d)	0.83***	0.87***							
LA Seedling^−1^ (21d)	0.63***	0.66***	0.82***						
LW 21d	0.66***	0.71***	0.82***	0.77***					
SLA 14d	0.67***	0.64***	0.65***	0.56***	0.60***				
SLA 21d	−0.01	−0.04	0.06	0.39***	−0.25*	−0.07			
LAI 21d	0.90***	0.91***	0.92***	0.87***	0.78***	0.66***	0.16***		
Plant DW (14d)	0.56***	0.63***	0.81***	0.70***	0.74***	0.24*	0.08	0.66***	
Plant DW (21d)	0.59***	0.64***	0.77***	0.76***	0.94***	0.56***	−0.24*	0.74***	0.68***

*significant at p < 0.05, *** significant at p < 0.001;

**SVI**: Seedling Vigor Index; **LA**: Leaf Area; **LW**: Leaf Weight; **SLA**: Specific Leaf Area; **LAI**: Leaf Area Index; **Plant DW**: Plant Dry Weight.

### Physiological traits measurements from greenhouse experiments

#### Alpha amylase activity in seeds germinating under flooding

**Figure 4:**
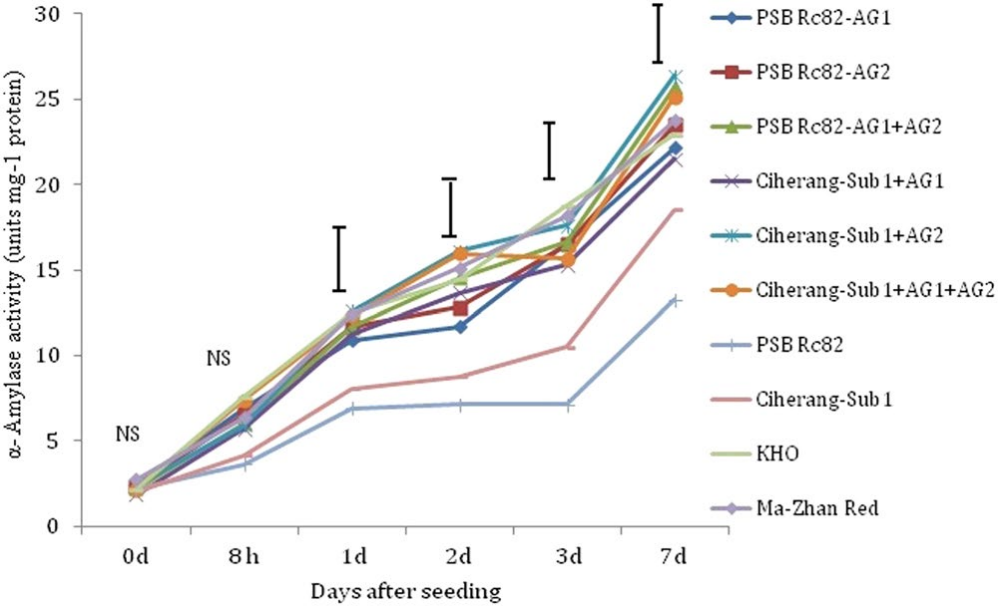
Activity of α-amylase (units mg^−1^ protein) in germinating seeds under 10 cm flooding during the first 7 days after seeding in the greenhouse experiment. Vertical bars indicate HSD_0.05_ (n = 60).

Alpha amylase activity was significantly higher in *AG1* and *AG2* introgressed lines along with their donor parents from 8 h to 7 DAS under flooded conditions (Fig. 4). Low α-amylase activity was observed in the dry seeds of all genotypes and no significant differences were found among genotypes. α-amylase activity increased progressively with time in seeds of tolerant genotypes germinating under flooded conditions until 7 DAS. The increase in activity was slower in the sensitive genotype PSB Rc82. In *AG1, AG2* and *AG1* + *AG2* introgressed lines, α-amylase activity was almost 50% higher than in dry seeds, but remained unchanged in the sensitive checks within 0 to 8 hours from seeding. Activity of α-amylase increased substantially from 8 h to 3 DAS, in almost all genotypes. Ciherang-Sub1-AG2, PSB Rc82-AG1-AG2 and Ciherang-Sub1-AG1-AG2 had relatively higher α-amylase activity at 7 DAS, while the lowest values were noted in the intolerant checks PSB Rc82 and Ciherang-Sub1 (Fig. 4).

### Non-structural carbohydrate in seeds germinating in flooded soil

**Figure 5:**
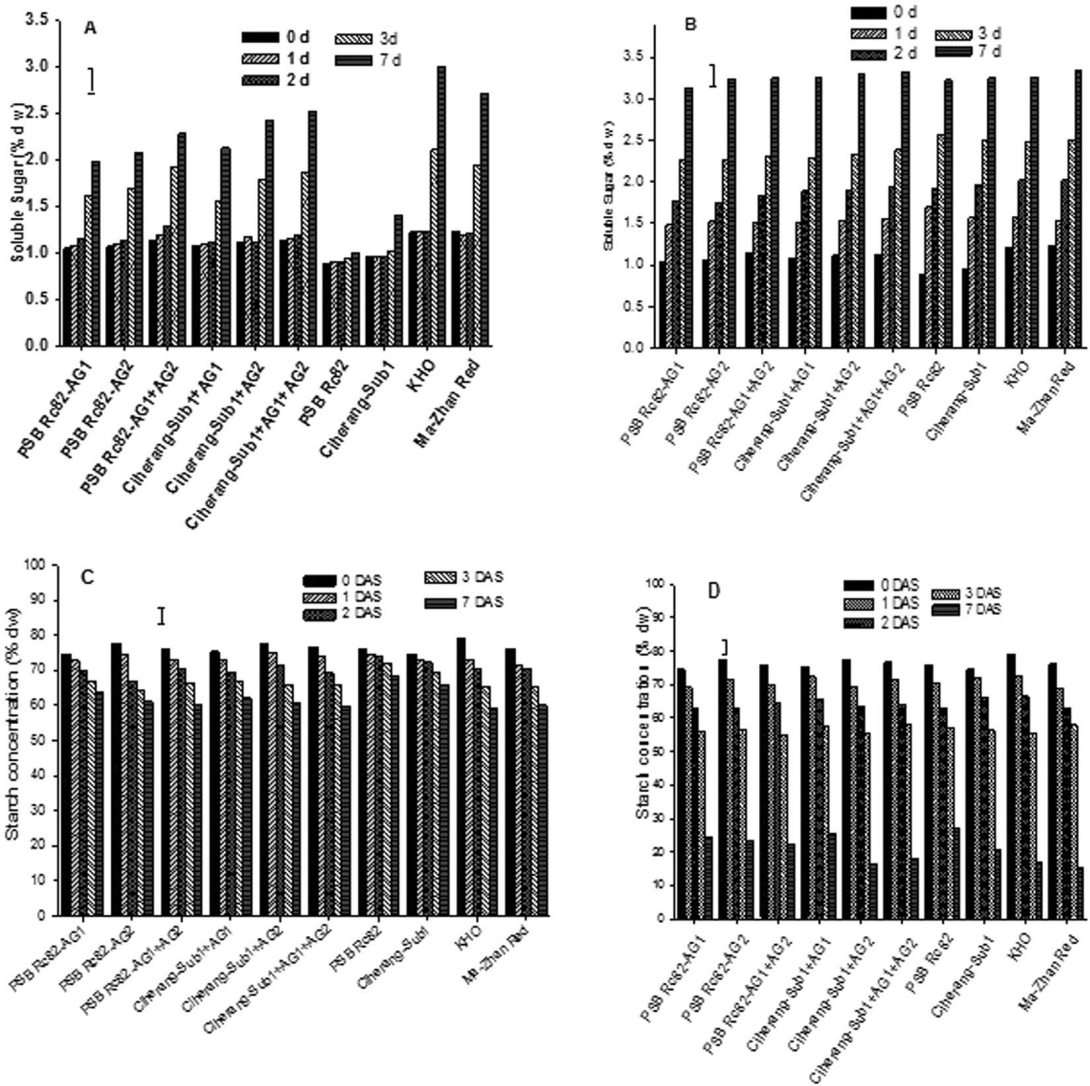
Soluble sugars (**A**) Flood, (**B**) control), and starch (**C**) Flood, (**D**) control) concentrations in germinating seeds under 10 cm flooding and control during the first 7 d after seeding in the greenhouse experiment. Vertical bars indicate HSD_0.05_ (n = 60).

Soluble sugar concentration in germinating seeds under flooded and control conditions was assessed within the first 7 DAS (Fig. 5). Flooding considerably reduced soluble sugar concentrations in germinating seeds during the first 0–2 DAS, which then progressively increased afterwards until 7 DAS in AG-introgression lines along with KHO and MR. In contrast, soluble sugar concentration in the sensitive genotypes decreased steadily from 0 to 7 DAS under flooded conditions, and only slightly increased at 3 DAS in Ciherang-Sub1 (Fig. 5A). Under control condition, soluble sugars increased similarly in both tolerant and sensitive genotypes from 0 to 7 DAS (Fig. 5B).

Starch degradation was slower in all genotypes under flooded conditions compared with the control, and was relatively faster in the tolerant AG lines (Fig. 5C,D). The tolerant checks as well as *AG2* and *AG1*-*AG2* introgression lines showed higher starch depletion during the period relative to the other rice genotypes. This reduction in starch concentration in germinating seeds was coupled with the increase in soluble sugars and subsequent faster seedling growth in the tolerant genotypes under flooded conditions.

### Non-enzymatic antioxidants and lipid peroxidation in germinating seeds under flooding

**Figure 6:**
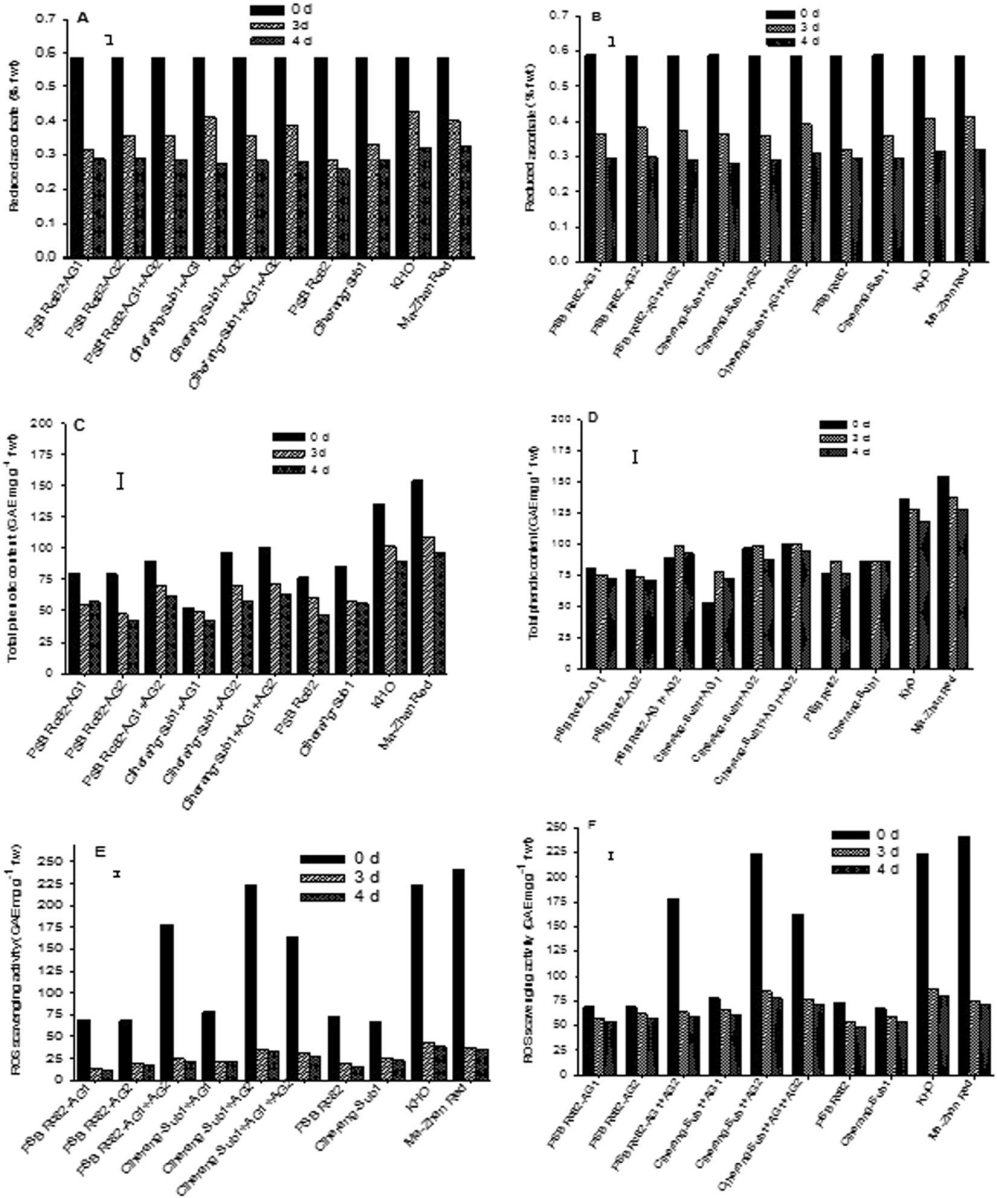
Reduced ascorbate (**A**) Flood, (**B**) control), total phenolic content (**C**) Flood, (**D**) control) and reactive oxygen scanvenging (ROS) scavenging activity (**E**) Flood, (**F**) control) in seeds germinating under 10 cm flooding; and control during the first 4 days after sowing in the greenhouse experiment. Vertical bars indicate HSD_0.05_ (n = 60).

Under flooded and control conditions, reduced ascorbate (AsA) decreased gradually from one DAS to 4 DAS, with proportionally greater reduction under flooded conditions (Fig. 6). A similar trend was observed for total ascorbate in both tolerant and sensitive rice genotypes (data not shown). At 3 DAS, reduced ascorbate concentration was higher in the AG lines compared with their respective intolerant parental lines, PSB Rc82 and Ciherang-Sub1 under flooded condition (Fig. 6).

KHO and MR showed higher concentrations of TPC than all other genotypes regardless of dates of sampling, both under controlled and flooded conditions (Fig. 6C,D). Among the AG-introgression lines, Ciherang-Sub1-AG1-AG2 and PSB Rc82-AG1-AG2 had higher TPC than their respective non-AG lines (Fig. 6C,D). Under controlled conditions, little change in TPC was observed with time within genotypes from 1 DAS to 4 DAS. In contrast, under flooded condition TPC declined progressively from 0 to 4 DAS, with the reduction rate relatively slower from 3 to 4 DAS (Fig. 6C,D). ROS scavenging activity substantially decreased with time under both control and flooded condition, with relatively faster rate under control conditions (Fig. 6E,F). Under flooded conditions, the highest activity was found in the tolerant checks and Ciherang-Sub1-AG1-AG2 during 3–4 DAS (Fig. 6E,F). Lipid peroxidation negatively correlated with ascorbate, reactive oxygen species scavenging activity, and TPC, while ROS correlated positively with ascorbate and TPC concentrations (Table 2).

**Table 2:** Correlation coefficients for the associations of reduced ascorbate (RAsA), total ascorbate (TAsA), ratio of RAsA and TAsA, malondialdehyde (MDA), total phenolic content (TPC), and reactive oxygen species (ROS) scavenging activity(n = 60; df = 9).

Parameter	Correlation Coefficient (r)				
	RAsA	TAsA	RAsA:TAsA	MDA	TPC
Total Ascorbate (TAsA)	0.98***				
RAsA:TAsA	0.90***	0.81***			
Malondialdehyde (MDA)	−0.70**	−0.67**	−0.68**		
Total Phenolic content (TPC)	0.36	0.34	0.39	−0.58*	
ROS scavenging activity	0.71***	0.69**	0.65**	−0.78***	0.69**

^*^significant at p < 0.05, ** significant at p < 0.01,*** significant at p < 0.001.

### Lipid peroxidation and reactive oxygen species scavenging activity

**Figure 7:**
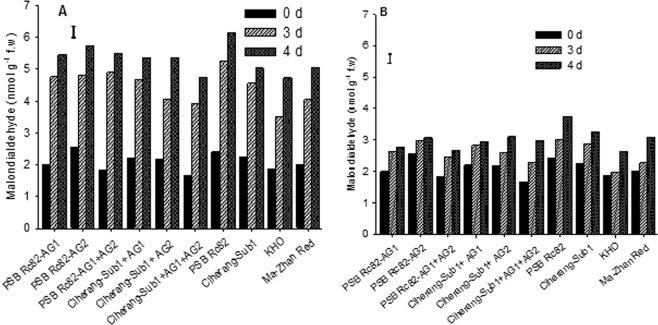
Concentration of malondialdehyde in germinating seeds under 10 cm flooding (**A**) and control (**B**) during the first 4 days after sowing in the greenhouse experiment. Vertical bars indicate HSD_0.05_ (n = 60).

Lipid peroxidation was estimated as the concentration of malondialdehyde (MDA) in germinating seeds during the first 4 days after seeding. MDA concentration progressively increased with time after sowing, under both flooded and control conditions. However, the increase was much more rapid under flooded conditions than the control (Fig. 7). Under flooding, the sensitive genotype PSB Rc82 had the highest amount of MDA at 3 and 4 DAS; whereas, Ciherang-Sub1-AG1-AG2 and the tolerant check KHO had lower concentrations of MDA at 4 DAS.

## Discussion

### Seedling emergence and seedling growth

Flooding impedes seed germination and early seedling growth especially when heavy rainfall occurs just after sowing or fields are not well leveled. Poor emergence and mortality of young seedlings caused by flooding result in poor crop establishment^1,3^. In this study, combining genetic tolerance with seed pre-treatments (hydro-priming or 24 hour soaking) increased seedling emergence (*P* < 0.001) and improved seedling growth under flooded condition^15^. The study further established the positive roles of combining *AG1* and *AG2* QTLs with proper seed treatments, which together had improved physiological process associated with seedling emergence and early seedling growth in flooded fields^3,41^.

Flooding after direct seeding negatively impacted seedling emergence with the consequent reduction in crop stand. Seedling emergence and biomass were higher in the AG introgression lines following flooding, with similar trends in both greenhouse and field trial. Stored starch was degraded and mobilized faster in the lines containing *AG1* and *AG2*, (Fig. 5), providing required energy for germination and seedling growth under flooded conditions^1,40,42–45^,. Comparatively, seedling emergence and early growth were poor in the sensitive genotypes (Fig. 3), as we also observed in a parallel study^7^. Earlier our greenhouse studies showed consistently higher seedling emergence of the AG lines under deeper flooding of up to 10 cm imposed after dry direct seeding^1,39^.

In the field experiment, seed priming and pre-soaking improved the emergence of seedlings under flooding (Fig. 1B,C), which agreed with previous reports^15,27^. Previous studies showed that seed pre-treatments shorten the time for seedling emergence, enhanced the seedling number, and accelerated seedling growth^16,46^. Our study showed that seedling emergence had significant positive correlation with SVI (r = 0.96, *P* < 0.001), LAI (r = 0.90, *P* < 0.001), plant biomass (Pearson correlation, r = 0.59, *P* < 0.001). Previously researchers also reported that, seedling emergence correlated positively with SVI^16,47^, leaf area index (LAI)^47,48^ and plant biomass production^15^. This has also been observed in other crops, including sorghum (*Sorghum bicolor*)^49^ and sunflower (*Helianthus annuus*)^23^. Seed pretreatment also accelerates pre-germination metabolism thus enhancing germination and crop establishment under favorable or stress conditions^17,50^. Seed hydro-priming induces activation and *de novo* synthesis of hydrolases that catalyze the breakdown of starch to sugar for translocation to the growing embryo^16^. In general, the positive effect of AG QTLs and seed pre-treatment was clearly reflected in the seedling emergence, growth and establishment of Ciherang-Sub1-AG1-AG2 and Ciherang-Sub1-AG2 (Fig. 1B,C). It has been shown that introgression of *SUB1* and AG QTLs have a positive effect on improving tolerance of flooding^41^, wherein AG QTLs are expressed during germination and early growth stages in flooded soils, while *SUB1* is expressed during vegetative stage and is effective up to heading stage^12,41,51^.

### Amylase activity and starch breakdown

In germinating seeds amylase enzymes catalyzes the breakdown of starch into maltose, with further hydrolysis into glucose via the enzyme glucosidase. Glucose then enters into the glycolytic pathway where it is used for the production of ATP and carbon molecules used for the plumule and radicle growth. In the present study, lower α-amylase activity was observed in all rice genotypes under flooded soil compared with control conditions. Germination of rice seeds in flooded soils and subsequent elongation of roots and shoots, has been associated with the ability to breakdown stored starch in the endosperm and use it for the growing embryo under hypoxia^1^. Soluble sugar concentration in seeds, and its translocation from the endosperm to the embryo, are closely associated with coleoptile growth under low or absence of oxygen^52,53^. The capability for sugar translocation from the endosperm to the coleoptile varies between rice cultivars, depending on ability to breakdown stored carbohydrates in the endosperm and the signaling mechanisms for supplying energy^42^.

Faster coleoptile elongation observed in Ciherang-Sub1-AG2, Ciherang-Sub1-AG1-AG2, and PSB Rc82-AG1-AG2, compared with Ciherang-Sub1and PSB Rc82, is due to the ability to maintain relatively higher soluble sugar concentrations as source of energy for coleoptile growth under hypoxic condition (Figs. 2, 4 and 5). AG introgression lines and the tolerant checks, KHO and MR, had higher emergence and higher α-amylase activity than the intolerant ones during flooding (Fig. 4). This result suggests a positive association between seedling emergence and α-amylase activity in germinating seeds under anaerobic conditions. This is further supported by the strong positive correlation between seedling emergence and α-amylase activity reported before^26^, with α-amylase gene (*RAmy3D*) found to have higher expression in germinating seeds of tolerant cultivars sown in flooded soil^1^. Here we observed that α-amylase activity was higher in Ciherang-Sub1-AG2, Ciherang-Sub1-AG1-AG2, PSB Rc82-AG1-AG2, and in the AG QTL donors KHO and MR during germination in flooded soil. In contrast, PSB Rc82 (sensitive) showed lower α-amylases activity under flooded condition, and consequently lower germination and emergence.

Soluble sugar in germinating seeds of all rice genotypes increased progressively from day 1 until 7 DAS under control condition (Fig. 5B); however, this increase was delayed until day 3 under flooded conditions (Fig. 5A). Similar but opposite trend was observed in starch breakdown, with slower starch mobilization in the intolerant genotypes in flooded soils (Fig. 5C,D). Clearly, the AG lines maintained relatively higher starch mobilization rates and higher sugar concentrations in germinating seedlings under flooding, leading to faster germination and early seedling growth, unlike the sensitive rice genotypes. The reduced rate of carbohydrate catabolism in these sensitive cultivars agreed with their slower rate of germination and growth in flooded soils, as also reported before^3,54^. The capacity to catalyze starch breakdown under hypoxic stress was associated with tolerance of anaerobic conditions in rice^52^. Recently the gene underlying tolerance at the *AG1* QTL locus was identified as *OsTPP7*^42^. This gene increases the sink strength of the growing embryo by maintaining signaling for low sugar availability, sustaining starch mobilization and sugar supply for growth^3,42^.

### Antioxidant properties and lipid peroxidation

Flooding leads to lipid peroxidation, which is one of the primary physiological processes indicative of the oxidative damage to cell structures where free radicals attack lipids containing carbon-carbon double bonds, ultimately leading to cell death^55,56^. Here we observed an association of physiological responses to early flooding with reduction in seedling emergence, increase in MDA concentration, and reduction in total phenolic content in rice seeds germinating in flooded soils. MDA concentration in germinating seeds increased substantially with the duration of flooding stress, with the highest concentration in the sensitive variety PSB Rc82 than in the AG-introgression lines and the tolerant checks KHO and MR (Fig. 7). Submergence stress was previously reported to be associated with the induction of oxidative stress in rice seedlings during the vegetative stage, with lower level of MDA content associated with submergence tolerance^37,57^.

A negative correlation was observed between MDA and total phenolic content in rice seeds germinating under flooded condition, suggesting the role of phenolic compounds in scavenging reactive oxygen species. The lower emergence of the sensitive genotypes is probably related in part, to the ROS induced lipid membrane damage as reflected in higher MDA content. Total phenolic content varied among the rice genotypes, with higher concentrations observed in Ciherang-Sub1-AG1-AG2 and the tolerant checks KHO and MR. The negative association between MDA and TPC, as an evidence for the role of phenolic compounds in free radical scavenging activity under stress conditions was also reported before^26^. Phenolic compounds are natural antioxidants that act as protective agents during stress and as scavengers of free radicals^58^. In red, brown or pigmented rice, MDA content is generally lower compared with white rice because of their higher phenolic content^59,60^. In our study we observed that, the de-husked grain of Ciherang-Sub1-AG1-AG2, Ciherang-Sub1-AG2, PSB Rc82-AG1-AG2, and the tolerant checks KHO and MR is brownish in color (Fig. S3), which is coupled with higher emergence and faster growth, as well as with other physiological traits positively associated with tolerance.

Ascorbate plays a vital role as an antioxidant in plant systems. Ascorbate can directly scavenge ROS through the ascorbate–glutathione cycle involving the enzyme ascorbate peroxidase; or non-enzymatically by reducing H_2_O_2_ directly to water^35^. Apart from its role as an antioxidant, ascorbate also acts as cofactor in some important enzymatic reactions for many metal-containing enzymes^61,62^. AsA is also induced during seed germination^63,64^ and if AsA concentration goes beyond 4 mM it inhibits seed germination^65^. Similarly, while AsA concentration in imbibed seeds in natural conditions is sufficient, its excess through internal production or exogenous application could aggravate the inhibitory effect of ABA on seed germination^64^. Apparently the AG-introgression lines and the tolerant checks maintained higher concentrations of total and reduced ascorbate compared with the sensitive checks (Fig. 6), and the correlation between ascorbate concentration and malondialdehyde content in germinating seeds under flooded condition is negative (Table 2). These results support the important role of ascorbate in reducing membrane damage during flooding stress, which is consistent with previous findings^37^.

## Conclusions

Rice genotypes carrying *AG1* and *AG2* QTls showed higher seedling emergence and faster elongation when dry seeds are sown in flooded soils, reflecting the important functional role of these QTLs in improving crop establishment under direct seeding. The introgression lines Ciherang-Sub1-AG2 and Ciherang-Sub1-AG1-AG2 had higher emergence and faster seedling growth than the other AG lines. Several traits were identified that are strongly associated with tolerance of anaerobic conditions during germination, including ability to degrade starch through maintenance of high α-amylase activity and subsequent increase in soluble sugars supply to the growing embryo; and maintaining lower MDA concentrations as an indication of cellular membrane integrity. The later was associated with higher concentration of reduced ascorbate, phenolic compounds, and reactive oxygen species (ROS) scavenging enzymes.

Seed hydro-priming as well as pre-soaking for 24 h further improved emergence and seedling growth of the AG introgression lines in flooded soils under field conditions. This suggests that combining genetic tolerance with proper seed management before sowing could effectively be used for direct seeding. Moreover, this approach can provide means for weed management, a major constraint for adoption of direct seeded systems in rice. Flooding the soil with 3–5 cm of water depth for the first 2–3 weeks after sowing can help control most of the weeds early in the season, as a cheap, effective and eco-friendly strategy for weed management in farmers’ fields^51^. The studies established the effectiveness of incorporating the AG QTLs in high yielding rice genotypes, and together with seed handling and pre-treatment, could ensure good crop establishment in flooded soils and encourage large-scale adoption of direct-seeded rice systems both in irrigated and rainfed ecosystems.

## Materials and Methods

### Plant materials and experiments

Experiments were conducted in a greenhouse and research field of the International Rice Research Institute (IRRI) in the Philippines. Greenhouse experiments were conducted to determine emergence percentage and physiological responses, while field experiments were conducted to assess agronomic performance of the contrasting genotypes.

#### Greenhouse experiments

Ten rice genotypes with anaerobic germination (AG) QTLs (quantitative trait loci), PSB Rc82-AG1, PSB Rc82-AG2, PSB Rc82-AG1-AG2, Ciherang-Sub1-AG1, Ciherang-Sub1-AG2, Ciherang-Sub1-AG1-AG2, along with their donors Kho Hlan On (*AG1*), Ma-Zhan Red (*AG2*) and the intolerant recipients PSB Rc82 and Ciherang-Sub1 were used in this study^7,41^. The experiment was conducted under control (non-flooded) and flooded condition during June 2016 using seedling trays. Each seedling tray had 34 rows, and each row had 17 cells (1 cm × 1 cm × 1.5 cm), filled with finely ground soil. One dry seed was sown per each of the 578 (34×17) cells half-filled with sterilized dry garden soil, and covered with 1 cm of fine soil. Fifty-one seeds were used per genotype, sown in three rows representing each replication. The trays were then placed in a concrete bench and flooded with tap water to a depth of 10 cm. Another set of trays was maintained under control (non flooded) condition in an adjacent bench^1,12^. The trial was laid in a randomized complete block design with three replications.

Growth parameters (seedling root and shoot length) were measured at 6, 10, 15, and 21 days after sowing (DAS) on 12 seedlings^48,66^. Seedling emergence percentage was calculated at 21 days after sowing based on the number of seedlings that emerged above the surface of floodwater following the below formula^1^.

$$Seedling\,emergence( \% )=\frac{Number\,of\,seedling\,emerged}{Number\,of\,seeds\,used\,per\,unit\,area}\times 100$$

Physiological traits including non-structural carbohydrates (starch and soluble sugars), α-amylase activity, concentrations of ascorbate and total phenolic compound, ROS scavenging activity, and lipid peroxidation were analyzed^1,12,48^. The methods and techniques used to measure the physiological traits are all well established and widely used, and they are just briefly described here with reference to relevant papers for more details.

### Measurement of alpha amylase activity

Alpha amylase activity was measured using Bernfeld’s method^67^, with slight modifications. About 200 mg of germinating seed sample was ground in liquid nitrogen using a mortar and pestle. Two mL of extraction buffer (100 mM HEPES-KOH pH 8.0, 5 mM MgCl2, 1 mM EDTA, 1 mM EGTA, 1 mM PMSF, 20% v/v glycerol, and 5 mM thiourea) were added to the samples followed by vortexing and agitating (4 **°**C) for 10 minutes and centrifuging at 10,000×g (4 °C) for 10 minutes. Aliquots of 200 μL crude plant extract were transferred into a plate with 50 μL 100 mM CaCl_2_, then heated at 70 °C for 15 minutes to deactivate β-amylase, and the heated extract was assayed for α-amylase activity.

One mL of 1% starch solution was added to the 200 μL each of the heated and unheated extract, and each sample was incubated at 20 **°**C for 0, 3, and 5 min to allow the conversion of starch to maltose, catalyzed by α-amylase. After incubation, 100 μL of 3,5-dinitrosalicylic acid color reagent was added to the solution. The samples were incubated in a boiling water bath for 3 min, cooled immediately in an ice bath and 1.8 mL ultra-pure water was added to each sample, vortexed, and absorbance read at 540 nm using a Spectrostar-Nano (BMG-LABTECH) spectrophotometer. The absorption values were compared with a standard curve for increasing amounts of maltose. The total protein concentration was determined using Bradford method^68^ with the activity expressed in units per milligram protein, and one unit of α-amylase activity defined as μ moles of maltose produced per minute.

### Measurements of soluble sugar and starch concentrations

Soluble sugars concentration was determined using the method of Fales^69^ with slight modifications. Approximately 30 mg of freeze-dried powdered sample was mixed with 1 mL of 80% ethanol, then vortexed and incubated in a water bath set at 75 °C for 10 minutes. Samples were then centrifuged at 5000×*g* for 10 minutes at 4 °C. The supernatant was collected and a second extraction performed with the residue using 1 mL 80% ethanol following same procedure. An aliquot of 30 μL of the stock sample was taken and diluted 1:50 to make a working sample, with a 30 μl diluted sample transferred to a plate containing 300 μL of anthrone reagent (0.4 g anthrone, 200 mL of concentrated sulfuric acid, 15 mL 95% ethanol, 60 ml of reagent grade water). The solution was vortexed and heated at 95 °C for 10 minutes followed by immediate cooling at 4 **°**C for 15 minutes and then normalized to room temperature. Absorbance was read at 620 nm using a Spectrostar-Nano (BMG-LABTECH) spectrophotometer, with soluble sugar concentration determined based on a standard curve for glucose.

Soluble sugar concentration was expressed as percent by weight of glucose per unit dry weight of the sample. After assaying soluble sugar, the residue was dried at 70 °C for 2 d and used for starch analysis following Ismail *et al*.^1^ with slight modifications. Starch was solubilized at 95 °C in hot water bath for 3 h with further hydrolysis using amyloglucosidase (Sigma Chemicals, St Louis, MO, USA) for 24 h. Absorbance was read at 450 nm using a Spectrostar-Nano (BMG-LABTECH) spectrophotometer, with starch concentration expressed as percent per unit dry weight of the sample.

### Ascorbate concentration

Ascorbic acid was determined following Shigeoka *et al*.^70^. Approximately 200 mg of fresh sample (germinating seeds) was ground to a fine powder with liquid nitrogen and extracted with 2 mL of 5% (w/v) metaphosphoric acid. The extract was clarified by centrifugation at 20 000 × *g* for 20 min at 4 **°**C. Two aliquots (each of 200 μL supernatant) were separately assayed for total ascorbate (AsA + DHAsA) and oxidized ascorbate (DHAsA). While 100 μL aliquot of 3 mM DCIP (as sodium salt of 2,6-dichloroindophenol) was added into the supernatant in the assay for total ascorbate, same volume of distilled water was added for the assay for oxidized ascorbate. After keeping the mixtures of both assays at room temperature for 20 min, aliquots of 200 μL of 1% (w/v) thiourea in 5% metaphosphoric acid and 200 μL of 10 mM DNPH (2,4-dinitrophenylhydrazine) was added. Mixtures were then incubated at 50 **°**C for 1 h, cooled in an ice bath for 15 min while adding 500 μL of ice-cold 85% (v/v) H_2_SO_4_. An aliquot of 200 μL of 20% (v/v) H_2_SO_4_ was added and absorbance was measured at 520 nm. Reduced Ascorbate concentration was calculated by subtracting oxidized ascorbate from total ascorbate.

### Total phenolic concentration (TPC) and reactive oxygen species (ROS) scavenging activity

The same supernatant extracted for lipid peroxidation was used for assaying total phenolic content^71^ and ROS scavenging activity^72^. Total phenolic content was determined spectrophotometrically using Folin-Ciocalteu’s phenol regent (Sigma Aldrich, Singapore). An aliquot of 100 μL supernatant or gallic acid standard was transferred into a plate with 200 μL Folin-Ciocalteu phenol reagent and vortexed, then incubated at 25 **°**C in darkness for 6 min. An 800 μL of 700 mM sodium carbonate solution was added and the mixture kept at 25 **°**C for 2 h in the dark. Absorbance was read at 765 nm using a Spectrostar-Nano (BMG-LABTECH) spectrophotometer. A standard curve was prepared using standard gallic acid (Sigma Aldrich, Singapore) solution, and the results were expressed as gallic acid equivalents (GAE g^−1^ fw).

To determine ROS scavenging activity, another aliquot of 100 μL supernatant or gallic acid standard was transferred to a plate with 6 μM 2,2-diphenyl-1-picrylhydrazyl (DPPH) and vortexed, then incubated at 25 **°**C for 30 min in the dark and absorbance read at 515 nm using a Spectrostar-Nano (BMG-LABTECH) spectrophotometer. A standard curve was prepared using standard gallic acid solution (Sigma Aldrich, Singapore), and the results were expressed as gallic acid equivalents (GAE g^−1^ fw).

### Determination of lipid peroxidation

Lipid peroxidation was determined by measuring malondialdehyde (MDA) formation using the thiobarbituric acid method^73^. First, 200 mg of germinating seeds were ground to a fine powder using liquid nitrogen and extracted with 2 mL of 0.01% trichloroacetic acid (TCA). The extracts were centrifuged at 4000 × *g* for 15 min at 4 **°**C, and the supernatant used for the assay. One mL aliquots of diluted samples were added into two separate test tubes. One mL of TBA solution consisting of 50.0% (w/v) trichloroacetic acid and 0.01% butylated hydroxytoluene was added to a test tube with 1 mL of aliquot. Another 1 mL of TBA solution composed of 50.0% (w/v) TCA and 0.65% TBA was added to the second test tube with 1 mL of aliquots. Samples were mixed thoroughly and heated at 95 °C for 25 min, then cooled and centrifuged at 4000×*g* for 10 min. Absorbance was read at 440, 532, and 600 nm using a Spectrostar-Nano (BMG-LABTECH) spectrophotometer. The malondialdehyde equivalent was calculated using the equations of Hodges *et al*.^73^.

#### Field experiment

Based on greenhouse screening experiments, we selected five genotypes—Ciherang-Sub1-AG1, Ciherang-Sub1*-*AG2, Ciherang-Sub1-AG1-AG2, Ma-Zhan Red (*AG2*) and Ciherang-Sub1 for field trial considering desired traits (anaerobic germination). Seeds of each genotype were subjected to three seed pre-treatments before sowing; (A) hydro-priming, (B) 24 h pre-soaking, and (C) dry seed (control seed). Following seeding, the field was flooded with 3–5 cm water depth or maintained saturated (control) using levees; laid in a split-plot design with three replications, where flood/control is main plot, and seed pretreatments are subplot. The trial was conducted twice, once each during the wet season (June-November) 2016, and the dry season (January-May) 2017.

For hydro-priming, seeds were soaked in aerated water for 24 h followed by air drying to 14% moisture (at room temperature, 25**°**C) and stored at 4 **°**C until use^15,47^. Seeds were hand-sown in a continuous line at 8 g m^−2^ in 10 cm × 10 cm spacing and 1 m ×1 m plots, then covered by 0.5 cm garden soil and flooded by 3–5 cm water depth for up to 21 d. Emerging seedlings were counted from a marked area of 0.25 m^2^ (5 rows of 0.5 m) at 4-d interval from seeding to 21 DAS. Seedling emergence (m^2^d^−1^) was calculated using the formula mentioned above in the  greenhouse experiment^1,74^. Seedling vigor index (SVI)^75^ was measured 21 DAS. Twelve seedlings/treatment from a marked area of 0.25 m^2^ at 14 and 21 DAS were uprooted (in flooded part where enough seedlings were absent adjusted to 12 seedlings) to measure leaf area, leaf number, root length, shoot length and dry weight (root + leaf+shoot). Area of detached green leaves was measured using leaf area meter (LiCor1 LI3100C). Leaf area index (LAI) was calculated as the total leaf area per unit land area^48^, and the specific leaf area (SLA)^48^ and specific stem biomass (SSB) were calculated, respectively, using the following formulae:

$${\rm{Specific}}\,{\rm{leaf}}\,{\rm{area}}(c{m}^{2}{g}^{-1})=\frac{Leaf\,area}{Leaf\,dry\,weight}$$

$${\rm{Specific}}\,{\rm{stem}}\,{\rm{biomass}}(mg\,c{m}^{-1})=\frac{Stem\,biomass\,}{Stem\,length\,}$$

### Environmental condition of greenhouse and field

In the greenhouse experiment, temperature was determined in greenhouse and floodwater at 1 cm and 5 cm water depth using digital thermometer (Model-8500-40, Chicago, IL 60648). Temperature was measured two times in a day, at 8:30am ranging from 26.1-34.6, 27.3-35.0, and 27.3-34.3 °C, respectively, and from 30.0-36.8, 32.1–38.2, and 32.1–38.1 °C, respectively at 1:30 pm (Fig. S1). The pH of floodwater was measured using Oakton-pH meter (Oakton pH11), water pH increased slight with increase in temperature and water depth, with the ranges of 7.6–8.8 (8:30am) and 7.9–9.0 (1:30 pm) at 1 cm depth, and 8.0–8.9 (8:30am) and 8.1–9.1 (1:30 pm) at 5 cm water depth (Table S1).

In the field experiment, temperatures were higher during the wet season of 2016 compared to the dry season of 2017. During the first 14 days after seeding, air temperatures ranged from 26.6–37.7 (2016) and 23.1–32.5 (2017), while water temperature ranged from 27.4–42.3 (2016) to 21.0–34.4 (2017). Mean photon flux densities (μmol m^−2^ s^−1^) was measured by LI-COR Quantum/Radiation/Photometer (LI-COR LI-250A). Mean photon flux densities (μmol m^−2^ s^−1^) above the water surface and at 3 cm water depth were 126.86–1307.8 and 88.27–885.17, respectively, in 2016 and 64.1–1256.1 and 34.5-899.0, respectively, in 2017 (Fig. S2). Water pH showed little variation with time and water depth, from 7.2–8.3 (2016) and 6.5–8.6 (2017).

### Statistical analysis

We conducted Two-way analysis of variance (ANOVA) test to evaluate the effect of two factors *genotypes* (factor 1) and *Flood and control conditions* (factor 2) simultaneously on the target traits. Q-Q Plots were used to test the assumption of normality and Bartlett’s test for homogeneity of variances was used. Analysis was conducted using STAR (Statistical Tool for Agricultural Research) for Windows version 2.0.1, which uses R package (IRRI, 2014). Sigma Plot 12.5 and Microsoft Excel 2016 were used for statistical computing and graphics analysis. Mean differences were compared by Tukey’s HSD (Honest Significant Difference) and LSD (Least Significant Difference) tests depending on the number of treatments in different experiments. Relationships between different attributes were determined using correlation analyses.

## Supplementary information

Supplementary information.
