# Supplementary material for: Responses of AG1 and AG2 QTL introgression lines and seed pre-treatment on growth and physiological processes during anaerobic germination of rice under flooding
Source: Sci Rep. 2020 Jun 23;10:10214. doi: 10.1038/s41598-020-67240-x (PMC7311552; doi:10.1038/s41598-020-67240-x)
Supplement: Supplementary file 1 — Supplementary information. [file 41598_2020_67240_MOESM1_ESM.pdf]

SUPPLEMENTARY DATA

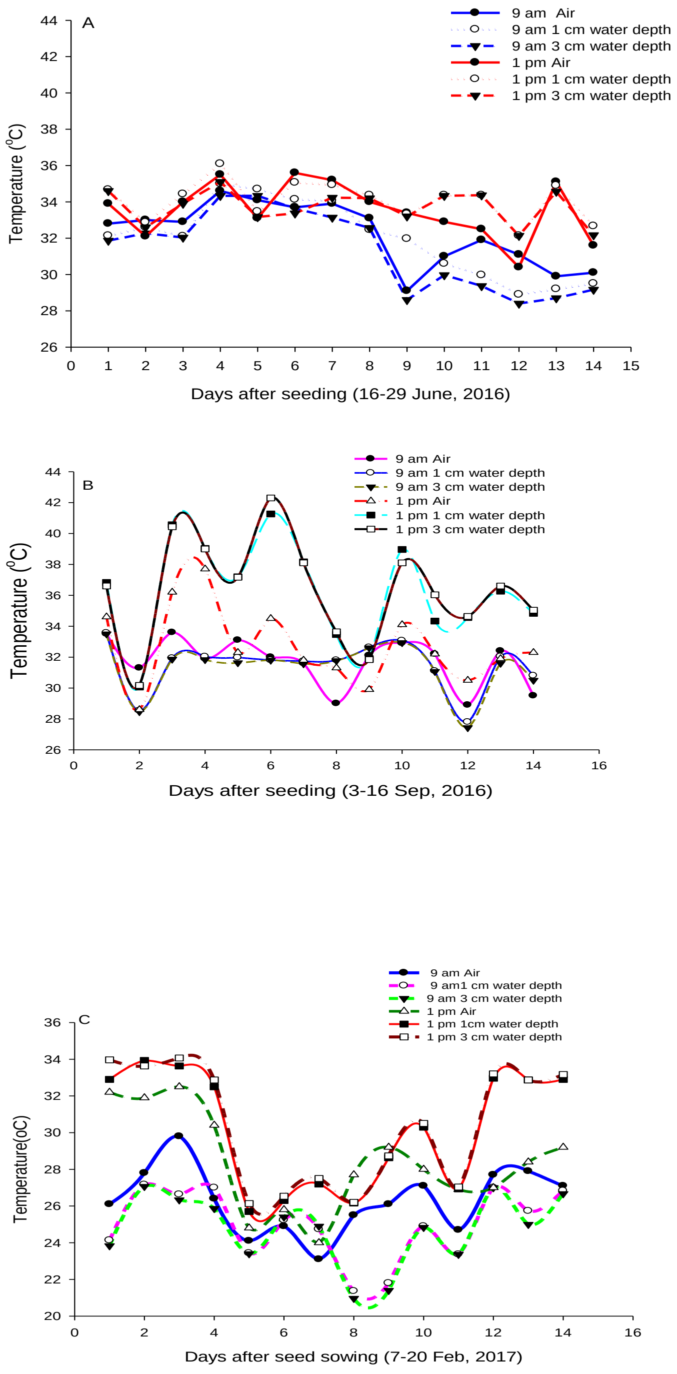

Figure S1. Air and water temperatures at 1 cm and 3 cm water depth during the first 14 days after seeding in the greenhouse (A) and field experiments conducted during 2016 WS (B) and 2017 DS (C) at IRRI research farm, Los Baños, Philippines.

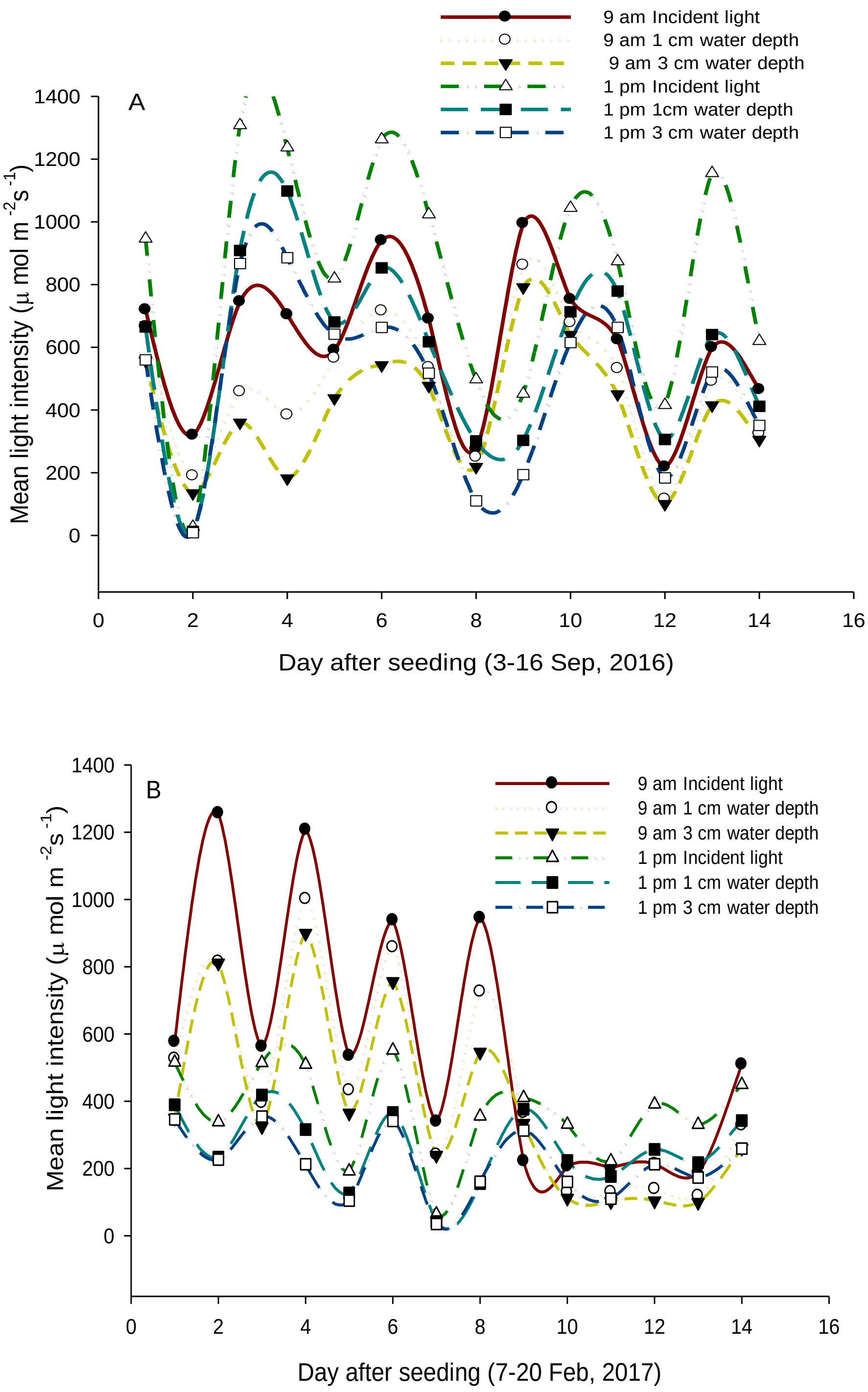

Figure S2. Mean light intensity (µ mol m^{-2} S^{-1}) in the air and at floodwater depths of 1 cm and 3 cm during the first 14 days after seeding in field experiments in 2016 WS (A) and 2017 DS (B), conducted at IRRI research farm, Los Baños, Philippines.

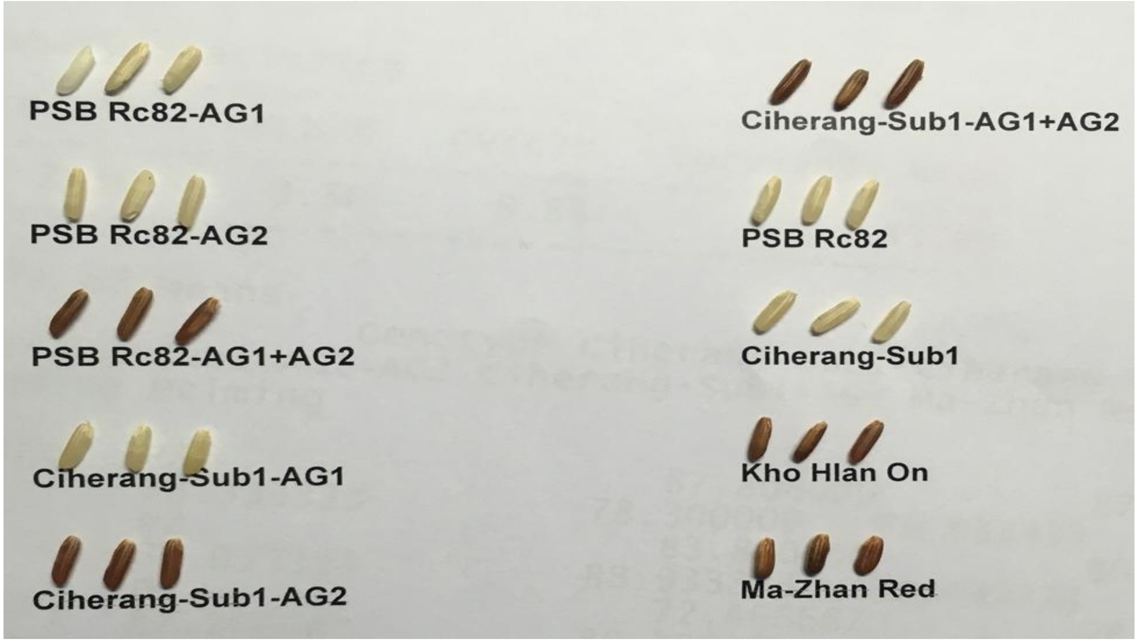

Figure S3. Representative de-husked seeds of the rice genotypes used in the greenhouse and field experiments.

Table S1

Water-pH at 1 cm and 3-5 cm water depth, in the greenhouse experiment during the wet season (WS) 2016 and field experiments during the 2016 WS and 2017 dry season (DS) at IRRI, Philippines.

**Time**	**Water pH**								
	**Greenhouse, 2016 WS**			**Field, 2016 WS**			**Field, 2017 DS**		
	**Date**	**1 cm**	**5cm**	**Date**	**1cm**	**5 cm**	**Date**	**1cm**	**5 cm**
8-9 am	16 June	7.6	7.9	03 Sep	7.5	7.6	07 Feb	7.1	7.0
1-2 pm		8.0	8.2		7.9	7.9		7.2	7.3
8-9 am	17 June	8.3	8.5	04 Sep	7.5	7.5	08 Feb	7.7	7.7
1-2 pm		8.2	8.1		7.7	7.7		8.5	8.5
8-9 am	18 June	8.3	8.5	05 Sep	7.2	7.2	09 Feb	8.1	8.2
1-2 pm		8.5	8.7		7.6	7.6		8.4	8.5
8-9 am	19 June	8.1	8.5	06 Sep	7.6	7.6	10 Feb	7.1	7.2
1-2 pm		8.7	8.9		7.5	7.5		7.0	6.9
8-9 am	20 June	8.0	8.0	07 Sep	7.7	7.7	11Feb	7.7	7.6
1-2 pm		8.8	9.1		8.0	8.0		7.4	7.4
8-9 am	21June	8.4	8.7	08 Sep	7.9	7.9	12 Feb	7.6	7.6
1-2 pm		8.7	8.9		8.0	8.0		7.6	7.6
8-9 am	22 June	8.8	9.0	09 Sep	7.8	7.8	13 Feb	7.7	7.8
1-2 pm		8.3	8.5		8.1	8.1		7.7	7.6
8-9 am	23 June	7.9	8.1	10 Sep	7.8	7.8	14 Feb	6.5	6.5
1-2 pm		8.2	8.5		7.6	7.7		7.2	7.2
8-9 am	24 June	8.2	8.3	11 Sep	7.9	7.8	15 Feb	8.5	8.6
1-2 pm		8.6	8.8		7.5	7.5		8.2	8.2
8-9 am	25 June	8.4	7.9	12 Sep	7.7	7.7	16 Feb	8.3	8.4
1-2 pm		8.6	8.8		7.9	7.9		7.9	7.8
8-9 am	26 June	8.5	8.7	13 Sep	7.9	7.9	17 Feb	7.3	7.3
1-2 pm		8.7	8.8		8.3	8.3		8.1	8.1
8-9 am	27 June	8.6	8.8	14 Sep	7.5	7.5	18 Feb	7.9	7.9
1-2 pm		8.6	8.8		7.9	7.9		7.7	7.6
8-9 am	28 June	8.3	8.5	15 Sep	7.6	7.7	19 Feb	7.9	7.9
1-2 pm		8.8	8.9		8.0	8.1		7.7	7.6
8-9 am	29 June	8.4	8.6	16 Sep	7.5	7.5	20 Feb	8.1	8.0
1-2 pm		8.7	8.8		8.0	8.1		7.4	7.3

Table S2.

Two-way ANOVA with three factors for “Seedling vigor index” using pre-treated seeds sown and flooded with 3-5 cm for 21 DAS, in the dry season 2017 field experiment.

Source of variations	df	SS	MS	F value	Pr(>F)
Genotype	4	8057491.86	2014372.97	60.7314483	0.0000000000
Flooding	1	179023567	179023567	5397.39199	0.0000000000
Priming	2	3383342.61	1691671.3	51.002297	0.0000000000
Genotype*Flooding	4	1884871.94	471217.985	14.2067786	0.0000000154
Genotype*Priming	8	308378.341	38547.2926	1.16216458	0.334295575
Residuals	70	2321797.22	33168.5317	NA	NA

Table S3.

Two-way ANOVA with three factors for “Leaf area per seedling” using pre-treated seeds sown and flooded with 3-5 cm for 14 DAS, in the dry season 2017 field experiment.

Source of variations	df	SS	MS	F value	Pr(>F)
Genotype	4	6.55847778	1.63961944	2.9218337	0.027021925
Flooding	1	163.94401	163.94401	292.15141	0.0000000000
Priming	2	32.15066	16.07533	28.6465503	0.0000000008
Genotype*Flooding	4	2.94476222	0.73619056	1.31190587	0.274061041
Genotype*Priming	8	6.51686222	0.81460778	1.45164688	0.190946172
Residuals	70	39.2812778	0.56116111	NA	NA

Table S4.

Two-way ANOVA with three factors for “Leaf area per seedling” using pre-treated seeds sown and flooded with 3-5 cm for 21 DAS, in the dry season 2017 field experiment.

Source of variations	df	SS	MS	F value	Pr(>F)
Genotype	4	152.191118	38.0477794	18.6925786	0.0000000002
Flooding	1	265.53409	265.53409	130.454837	0.0000000000
Priming	2	226.21766	113.10883	55.5694899	0.0000000000
Genotype*Flooding	4	41.5324378	10.3831094	5.10114105	0.00115117
Genotype*Priming	8	25.7595956	3.21994944	1.58193616	0.145973636
Residuals	70	142.481389	2.03544841	NA	NA

Table S5.

Two-way ANOVA with three factors for "Leaf weight" using pre-treated seeds sown and flooded with 3-5 cm for 21 DAS, in the dry season 2017 field experiment.

Source of variations	df	SS	MS	F value	Pr(>F)
Genotype	4	0.43606667	0.10901667	14.4344381	1.21E-08
Flooding	1	0.93025	0.93025	123.170488	4.39E-17
Priming	2	0.21606889	0.10803444	14.3043862	6.19E-06
Genotype*Flooding	4	0.13922222	0.03480556	4.60845716	0.0023208
Genotype*Priming	8	0.09145333	0.01143167	1.51361888	0.16822516
Residuals	70	0.52867778	0.00755254	NA	NA

Table S6.

Two-way ANOVA with three factors for "Specific leaf area" using pre-treated seeds sown and flooded with 3-5 cm for 14 DAS, in the dry season 2017 field experiment.

Source of variations	df	SS	MS	F value	Pr(>F)
Genotype	4	9921.98705	2480.49676	2.07608241	0.093136066
Flooding	1	127165.466	127165.466	106.432708	0.0000000000

Priming	2	15318.7923	7659.39617	6.41062626	0.0027763142
Genotype*Flooding	4	43203.005	10800.7513	9.0398222	0.0000060425
Genotype*Priming	8	40832.1015	5104.01269	4.27186647	0.000320965
Residuals	70	83635.7808	1194.79687	NA	NA

Table S7.

Two-way ANOVA with three factors for “Specific leaf area” using pre-treated seeds sown and flooded with 3-5 cm for 21 DAS, in the dry season 2017 field experiment.

Source of variations	df	SS	MS	F value	Pr(>F)
Genotype	4	40708.0572	10177.0143	13.2112088	0.0000000454
Flooding	1	1446.16659	1446.16659	1.87732946	0.1750165277
Priming	2	82050.2657	41025.1329	53.2564444	0.0000000000
Genotype*Flooding	4	9046.31181	2261.57795	2.93584912	0.0264711256
Genotype*Priming	8	22967.8261	2870.97827	3.72693721	0.0011103146
Residuals	70	53923.2263	770.331804	NA	NA

Table S8.

Two-way ANOVA with three factors for “Leaf area index” using pre-treated seeds sown and flooded with 3-5 cm for 21 DAS, in the dry season 2017 field experiment.

Source of variations	df	SS	MS	F value	Pr(>F)
Genotype	4	0.05789496	0.01447374	7.36085308	0.0000523310
Flooding	1	1.0989225	1.0989225	558.874741	0.0000000000
Priming	2	0.14579549	0.07289774	37.0733223	0.0000000000
Genotype*Flooding	4	0.02191011	0.00547753	2.78568499	0.033005936
Genotype*Priming	8	0.01673351	0.00209169	1.06376208	0.398352856

Residuals	70	0.13764189	0.00196631	NA	NA

Table S9.

Two-way ANOVA with three factors for "plant dry weight' using pre-treated seeds sown and flooded with 3-5 cm for 14 DAS, in the dry season 2017 field experiment.

Source of variations	df	SS	MS	F value	Pr(>F)
Genotype	4	82.6182956	20.6545739	11.308978	0.0000003902
Flooding	1	1268.40152	1268.40152	694.486608	0.0000000000
Priming	2	60.9141756	30.4570878	16.6761386	0.0000011946
Genotype*Flooding	4	17.0425178	4.26062944	2.33281815	0.064103082
Genotype*Priming	8	55.8579578	6.98224472	3.82298142	0.000890872
Residuals	70	127.847111	1.8263873	NA	NA

Table S10.

Two-way ANOVA with three factors for "plant dry weight" using pre-treated seeds sown and flooded with 3-5 cm for 21 DAS, in the dry season 2017 field experiment.

Source of variations	df	SS	MS	F value	Pr(>F)
Genotype	4	624.48804	156.12201	8.86305602	0.0000075433
Flooding	1	14836.62	14836.62	842.275822	0.0000000000
Priming	2	767.239607	383.619803	21.7781196	0.0000000443
Genotype*Flooding	4	73.7360222	18.4340056	1.04649962	0.389615018
Genotype*Priming	8	133.242093	16.6552617	0.94552022	0.485380861
Residuals	70	1233.0443	17.6149186	NA	NA
